# *Novadessus viracocha*, a new genus and species of Bidessini Sharp from Peru (Coleoptera, Adephaga, Dytiscidae, Hydroporinae)

**DOI:** 10.3897/zookeys.623.10018

**Published:** 2016-10-11

**Authors:** Kelly B. Miller

**Affiliations:** 1 Department of Biology and Museum of Southwestern Biology, University of New Mexico, Albuquerque, NM 87131-0001 USA

**Keywords:** Water beetles, taxonomy, classification, Neotropical, Novadessus, new genus, Dytiscidae, Coleoptera

## Abstract

*Novadessus viracocha* **gen. n.** and **sp. n.** is described from Peru. The genus distinctly is characterized by having the following combination: (1) a transverse occipital line absent on the head; (2) the anterior clypeal margin not modified; (3) a pair of basal pronotal striae present; (4) the basal elytral stria absent; (5) the elytral sutural stria absent; (6) the elytron without longitudinal carinae; (7) the epipleuron without a transverse carina at the humeral angle; (8) the lateral lobes of the male aedeagus two-segmented; (9) the overall habitus elongate and oval, with lateral pronotal and elytral margins discontinuous; (10) without distinct denticles along the posterior margins of the abdominal sternites; (11) the male genitalia (both median lobe and lateral lobes) bilaterally symmetrical; and (12) the metatrochanter small relative to the metafemur, approximately 0.6 × the length of the metafemur. The genus is diagnostically similar to *Fontidessus* Miller and Spangler and *Neobidessodes* Hendrich and Balke, but is superficially more similar to *Liodessus* Guignot. The habitus and male genitalia are illustrated, and a distribution map is provided.

## Abstract (es)

*Novadessus viracocha* **gen. n.** y **sp. n.** se describe de Perú. El género se caracteriza especialmente por tener la siguiente combinación: (1) línea occipital transversal ausente en la cabeza; (2) margen anterior clipeal no modificado; (3) presencia de un par de estrías basales pronotales; (4) estría elitral basal ausente; (5) estría elitral sutural ausente; (6) élitro sin carenas longitudinales; (7) epipleuron sin carena transversal en el ángulo humeral; (8) lóbulos laterales del aedeagus del macho bisegmentados; (9) hábito alargado y oval, con márgenes laterales de pronoto y élitros discontinuos; (10) sin dentículos a lo largo de los márgenes posteriores de los esternitos abdominales; (11) órganos genitales del macho (lóbulo medio y lóbulos laterales) bilateralmente simétricos; y (12) metatrocánter pequeño en relación con el metafémur, aproximadamente 0.6 × de la longitud del metafémur. El género es diagnósticamente similar a *Fontidessus* Miller y Spangler y *Neobidessodes* Hendrich y Balke, pero es superficialmente similar a *Liodessus* Guignot. Se ilustran el hábito y los órganos genitales del macho, y se proporciona un mapa de distribución.

## Introduction

The diving beetle tribe Bidessini Sharp has provided large numbers of new species and genera over the past few years, especially in the Neotropical region (Miller 2016; Miller and Garcia 2011; Miller and Montano 2014; Miller and Short 2015; Miller and Spangler 2008). The few characters that typically characterize bidessine genera come in numerous combinations making diagnoses complex and relationships difficult to establish. Bidessini is currently the largest clade of diving beetles, and the group promises to continue to grow as new diversity is discovered.

A new species was discovered among legacy specimens from the US National Collection that could not be assigned to an existing genus. Therefore, the goal of this project is to describe a new genus and new species of Bidessini from Peru.

## Materials and methods

**Measurements.** Measurements were taken using a Zeiss Discovery V8 dissecting microscope with an ocular scale. All known specimens were measured. Measurements include: 1) total length (TL), 2) greatest width across elytra (GW), 3) greatest width of pronotum (PW), 4) greatest width of head (HW), 5) distance between eyes (EW), 6) greatest length of metafemur (FL), and 7) greatest width of metafemur (FW). The ratios TL/GW, HW/EW and FW/FL were also calculated.

**Images.** Illustrations were made using a drawing tube on a Zeiss Discovery V8 dissecting scope. Sketches were first done in pencil then scanned, placed into an Adobe Illustrator artboard and “inked” digitally using vector lines.

## Results

### Novadessus gen. n.

Taxon classificationAnimaliaColeopteraDytiscidae

http://zoobank.org/15DD642E-6A90-4721-9C59-065C4A71F7EC

#### Type species.

*Novadessus viracocha* sp. n., by current designation.

#### Diagnosis.

*Novadessus* is characterized by the following combination: (1) a transverse occipital line absent on the head; (2) the anterior clypeal margin not modified; (3) a pair of basal pronotal striae present; (4) the basal elytral stria absent; (5) the elytral sutural stria absent; (6) the elytron without longitudinal carinae; (7) the epipleuron without a transverse carina at the humeral angle; (8) the lateral lobes of the male aedeagus two-segmented; (9) the overall habitus elongate and oval, with lateral pronotal and elytral margins discontinuous; (10) without distinct denticles along the posterior margins of the abdominal sternites; (11) the male genitalia (both median lobe and lateral lobes) bilaterally symmetrical; and (12) the metatrochanter small relative to the metafemur, approximately 0.6 × the length of the metafemur.

The genus is relatively similar in appearance and overall shape of the male genitalia to *Liodessus* Guignot, a rather generalized group of Bidessini species. However, *Novadessus* is missing both the transverse occipital line across the back of the head and the basal elytral striae, each of which is characteristic of *Liodessus*. In Miller and Bergsten (2016) the genus keys out to couplet 13 which separates *Fontidessus* Miller and Spangler and *Neobidessodes* Hendrich and Balke, neither of which is a convincing fit. *Fontidessus*, though Neotropical like *Novadessus*, have a characteristic ventral sclerite on the male median lobe and very large metatrochanters, neither of which are present in *Novadessus*. *Neobidessodes* have a rather different body shape (elongate oval with a continuous lateral body line), are Australian, and do not appear to be similar in any other particular way to *Novadessus*. *Spanglerodessus* Miller and García and *Amarodytes* Régimbart (both Neotropical) each also have the combination of absence of an occipital line, presence of pronotal striae and absence of elytral striae, but the first is very broad and broadly rounded laterally with a broad pronotal bead and the second has the pronotal striae generally short, curved and located somewhat laterally. In addition, each group is superficially quite distinct from *Novadessus* in many other less discrete characters such as body shape, overall coloration, surface sculpture, etc. Even so, the relationships of *Novadessus* to these other taxa, and to other Bidessini genera in general, is unknown and needs investigation.

#### Etymology.

This genus is named *Novadessus* from the Latin word, *novus*, meaning “new” and *dessus*, a common root for genera in the Bidessini.

### Modified key to Bidessini genera from Miller and Bergsten (2016)

**Table:** 

13a(12)	Body outline approximately continuously curved between pronotum and elytron (Miller and Bergsten fig. 37.13b)	**13**
13a’	Body outline discontinuous between pronotum and elytron (Fig. 1); Neotropical (Fig. 5)	***Novadessus* gen. n.**

**Figures 1–4.**
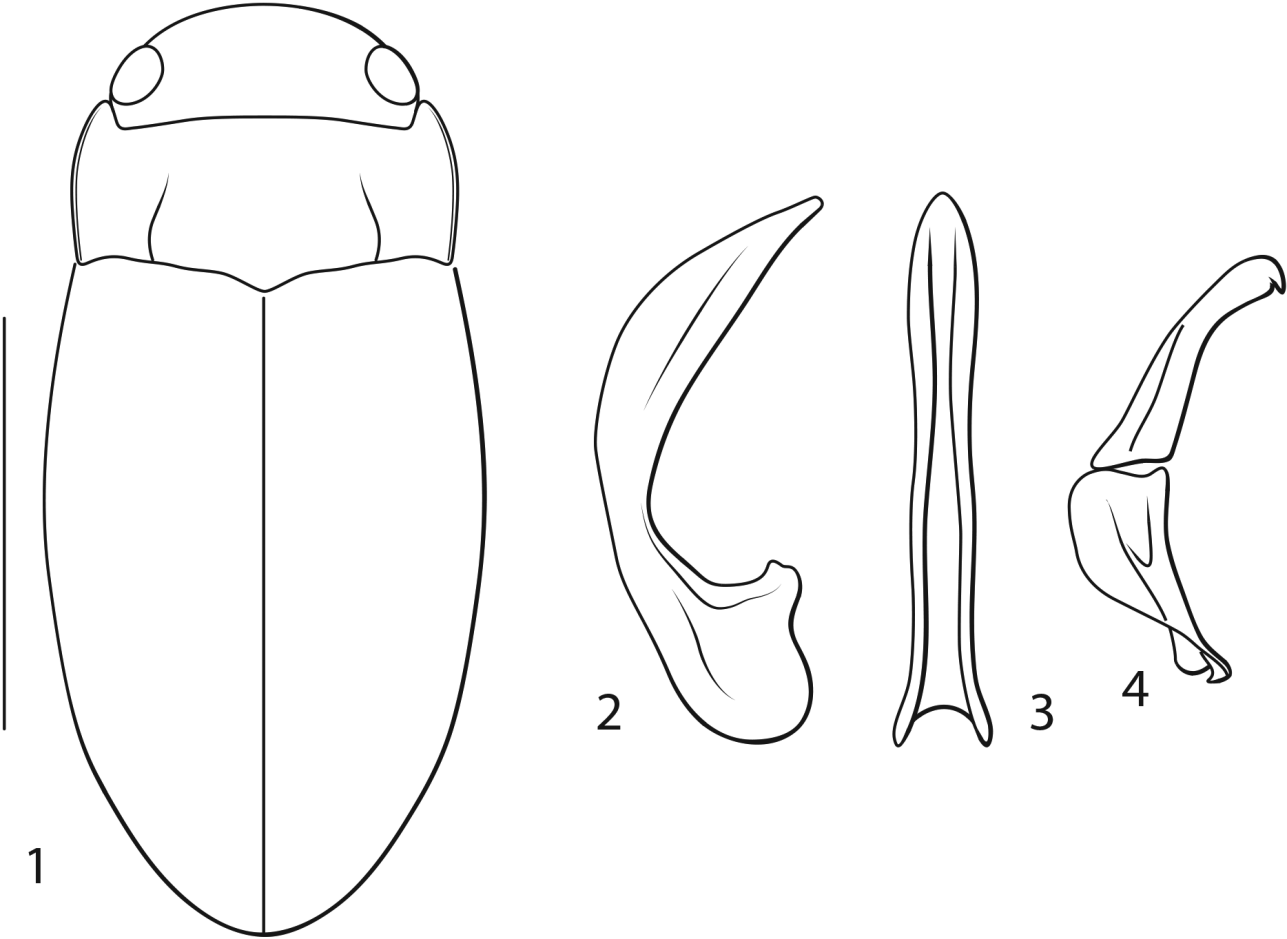
*Novadessus viracocha* sp. n. **1** Dorsal habitus, scale bar = 1.0 mm. **2–4** Male genitalia **2** Median lobe, right lateral aspect **3** Median lobe, ventral aspect **4** Right lateral lobe, right lateral aspect.

**Figure 5.**
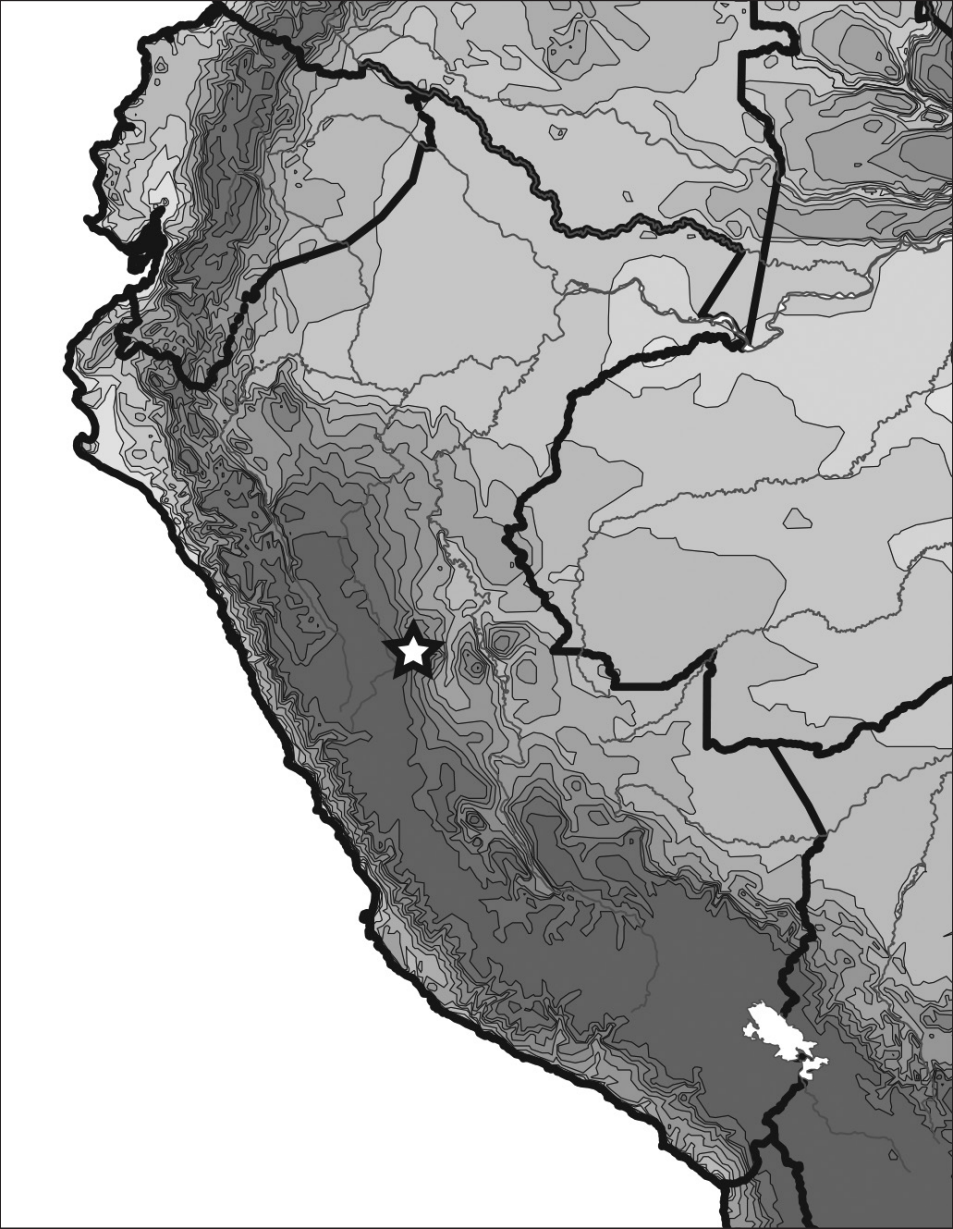
*Novadessus viracocha* sp. n. distribution.

### Novadessus viracocha

Taxon classificationAnimaliaColeopteraDytiscidae

Miller sp. n.

http://zoobank.org/7602EF1A-6DA2-4D90-9B2F-27ABBE96D550

Figs 1–4 , 5

#### Type locality.

Peru, Department Huanuco, Shishmay.

#### Diagnosis.

Monotypic. The male median lobe in lateral aspect is expanded medially and evenly curved to a narrowed, apically narrowly rounded apex (Fig. 2). The coloration is overall brownish.

#### Description.

*Measurements*. TL = 2.0–2.2mm, GW = 0.9–1.0mm, PW = 0.8–0.9mm, HW = 0.6–0.7mm, EW = 0.4–0.5mm, TL/GW = 2.1–2.2, HW/EW = 1.4–1.5. Body elongate oval, lateral outline somewhat discontinuous between pronotum and elytron (Fig. 1).

*Coloration*. Head, including appendages, evenly brown. Pronotum brown, somewhat darker along anterior and posterior margins. Elytron brown, lighter brown laterally. Ventral surfaces brown, legs, head and epipleuron lighter brown than thoracic and abdominal sternites.

*Sculpture and structure*. Head surface finely but distinctly microreticulate and micropunctate; without occipital line or modifications to evenly rounded clypeal margin. Pronotum surface smooth and shiny with few micropunctures scattered across surface; lateral margins broadly rounded with bead narrow; broadest anteriorly, narrowed posteriorly; lateral striae distinctive, extending anteriorly more than half distance across pronotum. Elytron smooth and shiny with fine, distinctive punctures distributed evenly; lateral margins subparallel anteriorly, narrowed to posteriorly pointed apex; basal and sutural striae absent. Prosternum moderately broad; prosternal process narrow, apically sharply pointed, medially rounded. Mesoventrite and metacoxal surfaces smooth and shiny with few, scattered fine punctures; metacoxal lines anteriorly somewhat divergent. Abdominal surfaces smooth and shiny with few scattered punctures. Metatrochanter small, ventrally rounded, not strongly offset from line of metafemur; metafemur slender, unmodified.

*Male genitalia*. Median lobe in lateral aspect evenly curved, medially distinctly and broadly expanded, apically narrowed to elongate, narrowly rounded apex (Fig. 2); in ventral aspect slender with lateral margins somewhat sinuate to narrowly rounded apex (Fig. 3). Lateral lobe with apical segment elongate slender with small ventral hook apically; ventral segment shorter, moderately broad (Fig. 4).

*Variation*. Specimens vary somewhat in intensity of coloration, but are otherwise similar.

#### Etymology.

This species is named *viracocha* after the Inca creator god.

#### Distribution.

The species is known from two series. The type series bears label data indicating it is from “Vic. of Shishmay.” Shishmay is a small town in the Andes of Peru (Fig. 5). The other series has label data indicating “Vic. San Domingo,” in Huanuco, Peru. This second locality could not be located.

#### Habitat.

The type series was collected from “highland lakes.” Nothing else is known of the habitat of *Novadessus viracocha*.

#### Type material.

Holotype in United States National Entomological Collection, Smithsonian Institution (USNM), male labeled, “Peru, S. A. Sept. 15-20, 1937 F. Woytkowski. No. 3787/ Dept. of Huanuco Vic. of Shishmay Andes 3600-4100 m Highland Lakes/ HOLOTYPE *Novadessus viracocha* Miller, 2016 [red label with black line border].” Paratypes (in USNM and The Museum of Southwestern Biology, University of New Mexico), 4 labeled same as holotype. Nine labeled, “Peru, S. A. Nov. 11-23, 1937 F. Woytkowski No. 3812/ Dept. Huanuco Vic. San Domingo Andes 3000 M. el./ PARATYPE *Novadessus viracocha* Miller, 2016 [blue label with black line border].”

## Supplementary Material

XML Treatment for Novadessus

XML Treatment for Novadessus viracocha
